# Fatal Arteriovenous Fistula Rupture in Renal-Predominant Eosinophilic Granulomatosis With Polyangiitis: A Case of Delayed Diagnosis

**DOI:** 10.7759/cureus.104476

**Published:** 2026-03-01

**Authors:** Minela Bećirović, Amela Alibegović, Emir Bećirović, Amir Bećirović

**Affiliations:** 1 Department of Nephrology, Clinic for Internal Medicine, University Clinical Center Tuzla, Tuzla, BIH; 2 Department of Hematology, Clinic for Hematology and Hematopoietic Stem Cell Transplantation, University Clinical Center Tuzla, Tuzla, BIH; 3 Intensive Care Unit, Clinic for Internal Medicine, University Clinical Center Tuzla, Tuzla, BIH; 4 Department of Endocrinology, Clinic for Internal Medicine, University Clinical Center Tuzla, Tuzla, BIH

**Keywords:** anca-associated vasculitis, arteriovenous fistula (avf), chronic kidney disease, eosinophilic granulomatosis with polyangiitis (egpa), hemorrhagic shock, rapidly progressive renal failure, vascular access complications

## Abstract

Eosinophilic granulomatosis with polyangiitis (EGPA) is a rare systemic vasculitis characterized by asthma, eosinophilia, and multisystem involvement. Renal manifestations are relatively uncommon but may be severe and rapidly progressive, and fatal hemorrhage from arteriovenous fistulas (AVFs) represents an uncommon yet catastrophic complication in patients with advanced kidney disease. We report a case of a 70-year-old man with long-standing asthma, chronic rhinosinusitis with nasal polyposis, marked eosinophilia, and progressive renal failure. After years of fragmented clinical manifestations, a clinical diagnosis of EGPA was considered based on clinical, laboratory, and immunological findings, supported by fulfillment of the 2022 American College of Rheumatology/European Alliance of Associations for Rheumatology (ACR/EULAR) classification criteria in the absence of histopathological confirmation, in the setting of rapidly progressive renal dysfunction. Induction immunosuppressive therapy with high-dose corticosteroids and cyclophosphamide was initiated. Due to advanced chronic kidney disease and the anticipated need for renal replacement therapy, a left radiocephalic AVF was constructed. Seventeen days later, the patient experienced spontaneous fistula rupture at home, resulting in massive hemorrhage, refractory hemorrhagic shock, and death. This case illustrates the consequences of delayed EGPA diagnosis and highlights the possibility of fatal vascular access complications in the setting of active systemic vasculitis, underscoring the importance of careful timing of invasive procedures, heightened clinical vigilance, and structured patient education when planning vascular access in patients with active inflammatory disease.

## Introduction

Eosinophilic granulomatosis with polyangiitis (EGPA), formerly known as Churg-Strauss syndrome, is a rare antineutrophil cytoplasmic antibody (ANCA)-associated vasculitis characterized by asthma, peripheral and tissue eosinophilia, and necrotizing inflammation of small- and medium-sized vessels [1]. The disease typically evolves through a prolonged prevasculitic phase dominated by allergic and eosinophilic manifestations, which may precede overt systemic vasculitis by several years and frequently result in delayed or missed diagnosis [2].

Renal involvement in EGPA is less common than in other ANCA-associated vasculitides but may be clinically significant when present [3]. It most often manifests as pauci-immune necrotizing glomerulonephritis and can follow an aggressive course, leading to rapid loss of kidney function and poor renal outcomes. In rare cases, renal involvement may represent the predominant clinical manifestation, complicating timely recognition of the underlying systemic disease [4]. Although prompt initiation of immunosuppressive therapy is essential in severe disease, a subset of patients progresses to end-stage kidney disease, necessitating early consideration of renal replacement strategies [5].

Hemodialysis vascular access represents a critical component of care in advanced kidney disease [6]. Although fatal hemorrhage from arteriovenous fistulas (AVFs) is rare, it is a well-documented and often sudden complication with devastating consequences [7]. However, the impact of active systemic vasculitis and immunosuppressive therapy on vascular access integrity remains poorly understood, particularly in the early postoperative period after vascular access creation [8].

Here, we report a case of renal-predominant EGPA complicated by fatal rupture of a newly constructed AVF shortly after vascular access creation. This case highlights the challenges of delayed diagnosis and underscores a potentially underrecognized risk associated with invasive vascular procedures performed during active systemic inflammatory disease. The diagnosis of EGPA may be supported by the 2022 American College of Rheumatology/European Alliance of Associations for Rheumatology (ACR/EULAR) classification criteria in the appropriate clinical context [9].

## Case presentation

A 70-year-old man was admitted to the Department of Nephrology, Clinic for Internal Medicine, University Clinical Center Tuzla, in February 2025 due to rapidly progressive deterioration of renal function accompanied by increasing fatigue and reduced exercise tolerance over the preceding three months. He denied dyspnea at rest, chest pain, fever, or infectious symptoms and reported preserved urine output proportional to fluid intake.

His medical history was notable for bronchial asthma diagnosed in 2018, treated with inhaled corticosteroids and long-acting bronchodilators. Serial spirometry demonstrated persistent airflow limitation consistent with chronic obstructive physiology. He also had a long-standing history of chronic rhinosinusitis with nasal polyposis and underwent functional endoscopic sinus surgery in August 2021. Histopathological analysis revealed chronic inflammatory nasal polyps without evidence of granulomatous inflammation or vasculitis.

In October 2021, the patient developed acute urinary retention associated with left-sided hydronephrosis. Prostate biopsy demonstrated chronic prostatitis with extensive eosinophilic infiltration. In March 2022, a repeat biopsy confirmed prostate adenocarcinoma, which was treated with definitive radiotherapy without adjuvant chemotherapy.

Renal function began to decline in late 2022, with serum creatinine first elevated to 118 µmol/L (estimated glomerular filtration rate (eGFR) ≈57.3 mL/min/1.73 m^2^, Chronic Kidney Disease Epidemiology Collaboration (CKD-EPI) 2021) [10]. Progressive deterioration followed, with levels rising to 243 µmol/L in November 2024 (eGFR ≈24.1 mL/min/1.73 m^2^), 465 µmol/L in January 2025 (eGFR ≈11.0 mL/min/1.73 m^2^), and 723 µmol/L at admission (eGFR ≈6.5 mL/min/1.73 m^2^). During this period, the patient developed normocytic anemia requiring transfusion support. He also reported intermittent bilateral hand paresthesias over approximately two years (Table 1), and subsequent neurological consultation supported the presence of chronic sensorimotor polyneuropathy.

**Table 1:** Chronological clinical events and their diagnostic relevance to EGPA EGPA, eosinophilic granulomatosis with polyangiitis; ENT, ear, nose, and throat; ANCA, antineutrophil cytoplasmic antibody; p, perinuclear; AVF, arteriovenous fistula.

Timepoint	Clinical event	Key findings	Diagnostic domain relevant to EGPA
2018	Diagnosis of bronchial asthma	Persistent airflow limitation; long-term inhaled corticosteroid therapy	Allergic phase (respiratory involvement)
2020-2021	Chronic rhinosinusitis with nasal polyposis	Functional endoscopic sinus surgery (Aug 2021); chronic inflammatory polyps	Upper airway involvement (ENT domain)
Oct 2021	Acute urinary retention with hydronephrosis	Prostate biopsy with eosinophilic infiltration	Extrapulmonary tissue eosinophilia
Mar-Jul 2022	Prostate adenocarcinoma	Biopsy-confirmed adenocarcinoma; definitive radiotherapy	Alternative cause of eosinophilia considered and excluded
Late 2022	Initial renal dysfunction	First creatinine elevation; emerging anemia	Possible early renal involvement
Mar 2023	Peripheral eosinophilia	Sustained elevation of eosinophil count	Hematologic domain (eosinophilic phase)
Nov 2024	Accelerated renal deterioration	Worsening kidney function and eosinophilia	Progression to vasculitic phase
Jan 2025	Rapid renal decline	Further renal impairment and anemia	Rapidly progressive renal involvement
Feb 2025	Hospital admission	Severe renal failure; p-ANCA positivity; elevated IgE	Immunologic domain (ANCA-associated vasculitis)
Feb 2025	Neurological evaluation	Chronic sensorimotor polyneuropathy	Peripheral nervous system involvement
Mar 2025	Imaging and diagnostic workup	Pleural and pericardial effusions; chronic renal damage	Multisystem involvement
Mar 4-11, 2025	Induction immunosuppressive therapy	High-dose corticosteroids and cyclophosphamide	Treatment for organ-threatening disease
Mar 6, 2025	AVF construction	Left radiocephalic AVF; uncomplicated course	Access created during the active vasculitic phase
Mar 17, 2025	Hospital discharge	Partial renal stabilization; preserved urine output	Pre-dialysis management
Mar 23, 2025	Fatal event	AVF rupture; hemorrhagic shock	Catastrophic vascular complication

On admission, the patient was afebrile, hemodynamically stable, and fully oriented. Cardiopulmonary examination revealed diffusely diminished breath sounds without wheezing and normal heart sounds without murmurs. No peripheral edema, cutaneous lesions, lymphadenopathy, or organomegaly were observed.

Laboratory evaluation demonstrated severe renal impairment, with serum creatinine peaking at 833 µmol/L (eGFR ≈5.5 mL/min/1.73 m^2^, CKD-EPI 2021) and urea levels reaching 59.4 mmol/L. Marked peripheral eosinophilia was present, with eosinophils accounting for up to 36.3% of leukocytes and an absolute eosinophil count of 4.64 × 10^9^/L (reference range: 0.00-0.43 × 10^9^/L). Review of prior laboratory results documented peak eosinophilia up to 5.74 × 10^9^/L during the pre-admission period. Hemoglobin reached a nadir of 73 g/L (reference range: 138-175 g/L), accompanied by transient thrombocytosis. Inflammatory markers were mildly to moderately elevated. Urinalysis revealed proteinuria and microscopic hematuria, while urine cultures remained sterile. Hypoalbuminemia (27 g/L; reference range: 32-46 g/L) and reduced total serum protein levels (52 g/L; reference range: 66-80 g/L) were also noted.

Immunological testing revealed positivity for perinuclear ANCA (p-ANCA) (64 U/mL; reference value: <5 U/mL) with increased serum IgE levels (248 IU/mL), while cytoplasmic ANCA (c-ANCA) was negative. Complement levels, rheumatoid factor, circulating immune complexes, anti-glomerular basement membrane antibodies, and infectious serologies were within reference ranges (Table 2).

**Table 2:** Laboratory and immunological findings at admission with corresponding reference ranges CRP, C-reactive protein; HPF, high-power field; ANCA, antineutrophil cytoplasmic antibody; p, perinuclear; c, cytoplasmic; GBM, glomerular basement membrane.

Parameter	Patient value	Reference range
Hemoglobin (g/L)	86	138-175
Platelet count (×10⁹/L)	481	158-424
Eosinophils (%)	36.3	0-7
Absolute eosinophil count (×10⁹/L)	4.64	0.00-0.43
Urea (mmol/L)	32.3	3.0-9.2
Creatinine (µmol/L)	734	71-115
Total serum protein (g/L)	52	66-80
Albumin (g/L)	27	32-46
CRP (mg/L)	9.9	<5.0
Urine protein	++	Negative
Urine erythrocytes (per HPF)	17	<4
Urine leukocytes (per HPF)	9	<5
Urine culture	Sterile	-
p-ANCA (U/mL)	64	<5
c-ANCA	Negative	-
IgE (IU/mL)	248	<100
C3 complement (g/L)	1.06	0.90-1.80
C4 complement (g/L)	0.183	0.10-0.40
Rheumatoid factor (IU/mL)	15	0-15
Circulating immune complexes C1q (RU/mL)	3.50	<20
Anti-GBM antibodies	Negative	-

**Figure 1:**
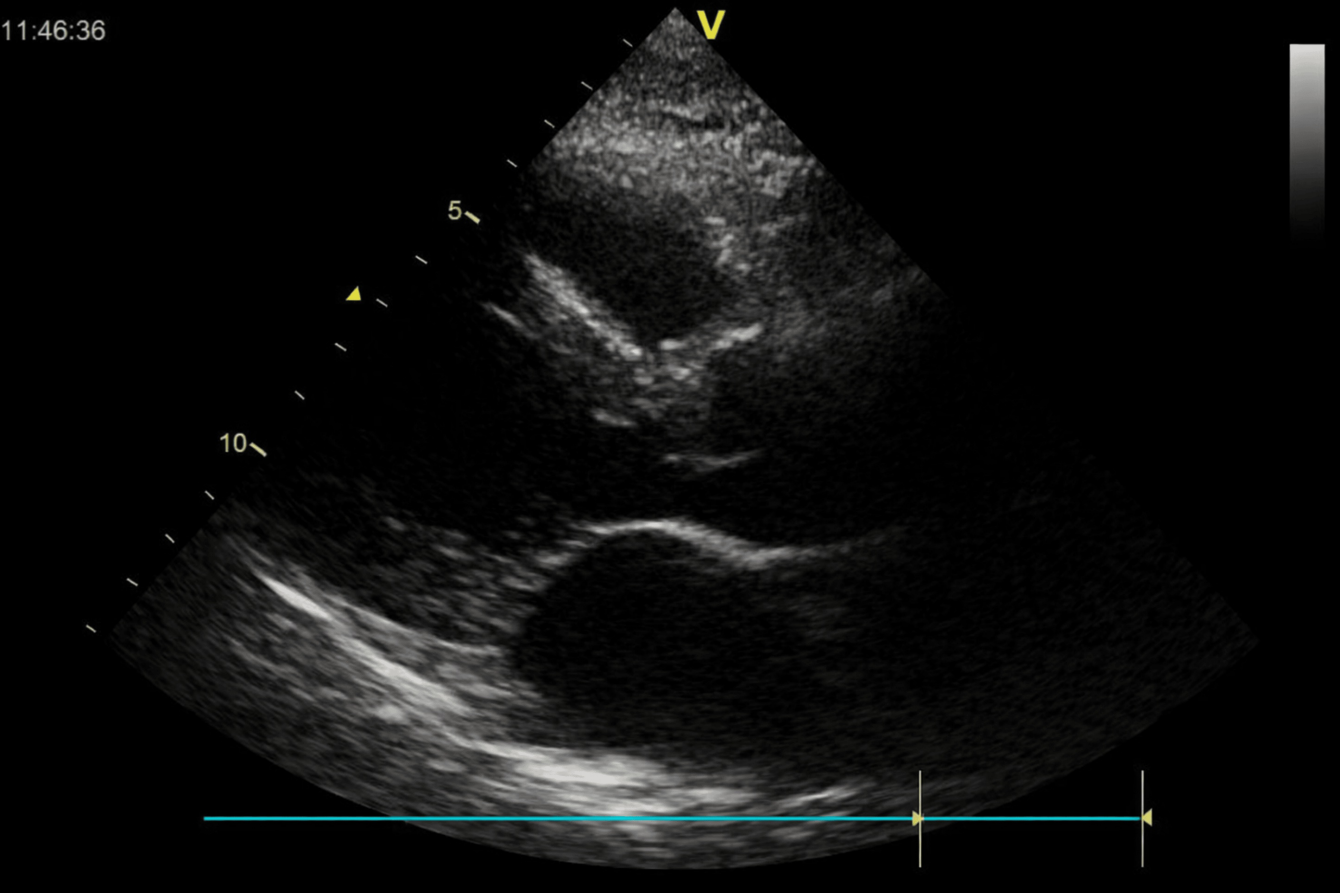
Transthoracic echocardiography findings Parasternal long-axis view demonstrating preserved left ventricular systolic function with mild concentric hypertrophy and no significant chamber dilation.

Transthoracic echocardiography demonstrated preserved left ventricular systolic function with mild concentric hypertrophy and mild mitral and tricuspid regurgitation (Figure 1).

**Figure 2:**
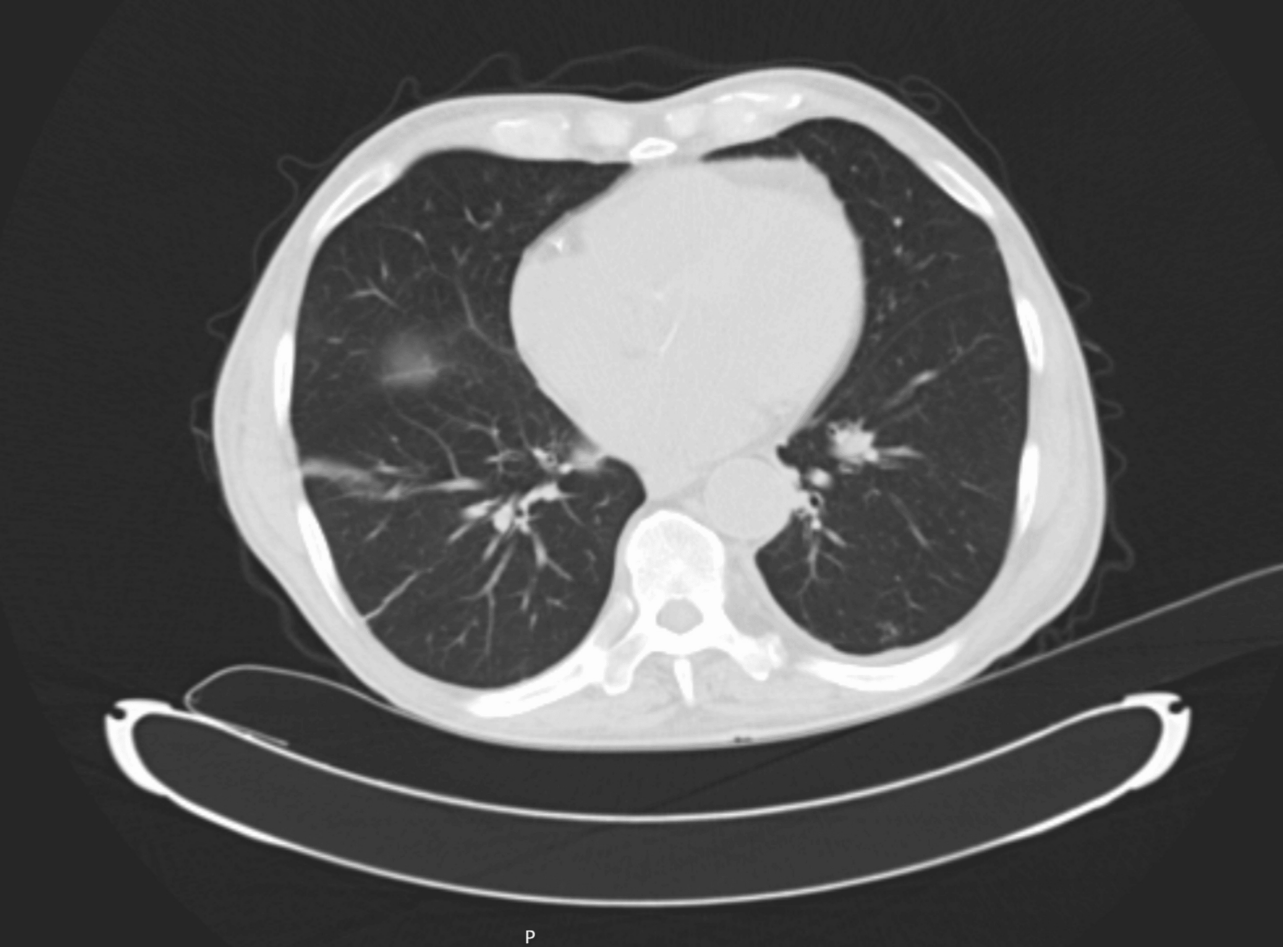
Chest computed tomography findings Axial chest computed tomography demonstrating pleuropulmonary fibrotic changes and pericardial effusion.

Chest computed tomography revealed bilateral pleural effusions, pleuropulmonary fibrotic changes, and a pericardial effusion measuring up to 21 mm, without mediastinal or axillary lymphadenopathy (Figure 2).

**Figure 3:**
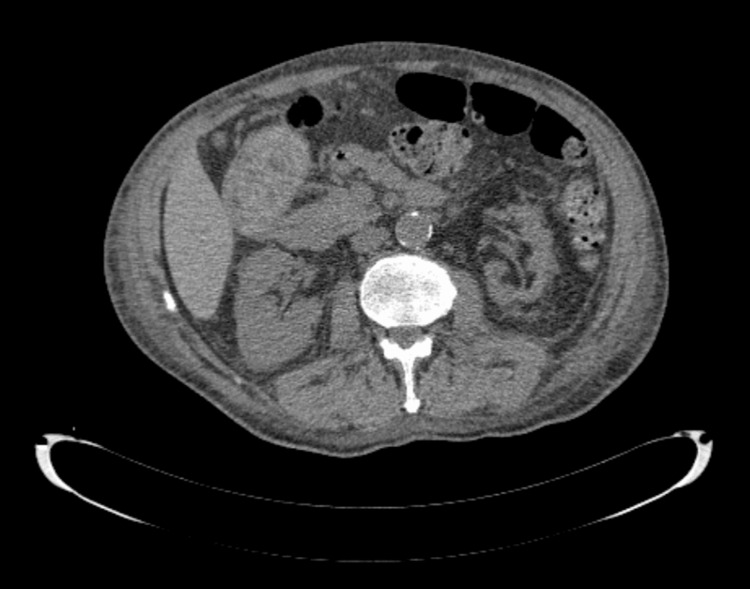
Abdominal computed tomography findings Axial abdominal computed tomography demonstrating bilateral reduction in renal cortical thickness consistent with chronic parenchymal damage.

Renal ultrasonography initially demonstrated hyperechogenic kidneys bilaterally with cortical thinning, consistent with chronic parenchymal damage. These findings were subsequently confirmed on abdominal computed tomography, which demonstrated bilateral reduction in renal cortical thickness; given the chronic imaging appearance, anticipated low diagnostic yield, and procedural risk in advanced kidney disease, renal biopsy was not pursued (Figure 3).

According to the available histopathology report, esophageal biopsies obtained during evaluation for suspected eosinophilic esophagitis demonstrated normal mucosa without eosinophilic infiltration. 

Based on the clinical, laboratory, and imaging findings, a multidisciplinary team concluded that EGPA with predominantly renal involvement was the most likely diagnosis, supported by the presence of adult-onset asthma, chronic rhinosinusitis with nasal polyposis, marked peripheral eosinophilia, p-ANCA positivity, elevated serum IgE levels, and chronic sensorimotor polyneuropathy. Primary hypereosinophilic syndrome and malignancy-related eosinophilia were excluded.

High-dose intravenous methylprednisolone pulse therapy was initiated and subsequently tapered, resulting in rapid normalization of peripheral eosinophil counts and partial stabilization of renal function, reflected by the absence of further increase in serum creatinine levels following treatment initiation and preserved urine output without the immediate need for renal replacement therapy. Cyclophosphamide was introduced as induction therapy. Given advanced chronic kidney disease and the anticipated need for renal replacement therapy, a left radiocephalic AVF was surgically constructed on March 6, 2025. AVF creation was performed in accordance with standard nephrology practice to facilitate timely pre-dialysis vascular access planning in patients with advanced chronic kidney disease. The procedure and early postoperative course were uncomplicated.

**Figure 4:**
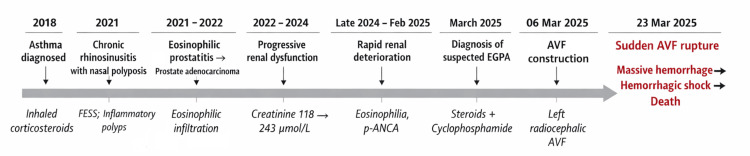
Chronological clinical timeline illustrating the progression from allergic and eosinophilic manifestations to renal-predominant EGPA, delayed diagnosis, initiation of immunosuppressive therapy, construction of an AVF during active systemic disease, and subsequent early fatal vascular access rupture. AVF, arteriovenous fistula; EGPA, eosinophilic granulomatosis with polyangiitis; FESS, functional endoscopic sinus surgery; ANCA, antineutrophil cytoplasmic antibody; p, perinuclear.

The patient remained clinically stable and was discharged on March 17, 2025, with preserved urine output and plans for continued immunosuppressive therapy and close nephrology follow-up, and was prescribed acetylsalicylic acid 100 mg daily at discharge, while prophylactic low-molecular-weight heparin administered during hospitalization following vascular access creation had been discontinued prior to discharge (Figure 4).

On March 23, 2025, 17 days after fistula construction, the patient experienced a spontaneous, sudden rupture of the AVF at home. Profuse bleeding ensued, and despite immediate compression and emergency transport, he arrived at the emergency department in profound hemorrhagic shock with agonal respiration and unmeasurable blood pressure. Advanced cardiopulmonary resuscitation was initiated but unsuccessful, and death was pronounced at 09:45. The immediate cause of death was hemorrhagic shock due to AVF rupture in the context of advanced renal failure and active systemic vasculitis. According to the 2022 ACR/EULAR classification criteria for EGPA, the patient achieved a total score of 16 points (≥6 required for classification), which supported the clinical classification of EGPA in the appropriate clinical context despite the absence of histopathological confirmation (Table 3) [9].

**Table 3:** Fulfillment of 2022 ACR/EULAR classification criteria for EGPA ACR/EULAR, American College of Rheumatology/European Alliance of Associations for Rheumatology; EGPA, eosinophilic granulomatosis with polyangiitis; ANCA, antineutrophil cytoplasmic antibody; p, perinuclear.

Criterion	Patient finding	Score
Obstructive airway disease (asthma)	Present since 2018	+3
Nasal polyps	Chronic rhinosinusitis with nasal polyposis	+3
Peripheral eosinophilia >1 × 10⁹/L	Peak absolute eosinophil count 5.74 × 10⁹/L	+5
Mononeuritis multiplex or polyneuropathy	Chronic sensorimotor polyneuropathy confirmed by neurological evaluation	+1
Positive p-ANCA	64 U/mL	+5
Hematuria	Microscopic hematuria present on urinalysis	-1
Total score	-	16 (≥6 required for classification)

It should be emphasized that the 2022 ACR/EULAR classification criteria are intended for research classification rather than standalone diagnosis; however, in the appropriate clinical context, fulfillment of these criteria may support clinical disease classification in the absence of histopathological confirmation [11].

## Discussion

This case highlights several clinically important aspects of EGPA, particularly the challenges of delayed diagnosis, the severity of predominantly renal involvement, and the potential risks associated with invasive vascular procedures performed during active systemic inflammation.

EGPA is characterized by a prolonged prevasculitic phase, often dominated by asthma, chronic rhinosinusitis, and tissue eosinophilia, which may precede overt vasculitis by several years [12]. In the present case, long-standing asthma, nasal polyposis, and eosinophilic infiltration of prostatic tissue were documented well before the development of rapidly progressive renal failure. These manifestations were initially interpreted as isolated organ-specific conditions rather than components of a unifying systemic disease, contributing to diagnostic delay [13]. This pattern reflects real-world clinical practice, in which fragmented presentations frequently obscure timely recognition until irreversible organ damage has occurred. In retrospect, the combination of adult-onset asthma, chronic rhinosinusitis with nasal polyposis, marked peripheral eosinophilia (peak absolute eosinophil count 5.74 × 10^9^/L), p-ANCA positivity (64 U/mL), elevated serum IgE (248 IU/mL), and neurologically confirmed chronic sensorimotor polyneuropathy was highly suggestive of a systemic eosinophilic vasculitic process, supportive of EGPA classification in the appropriate clinical context despite the absence of histopathological confirmation [14,15].

Renal involvement in EGPA is less frequent than in other ANCA-associated vasculitides but may follow an aggressive course once established [16]. The rapid progression from mildly elevated creatinine to advanced kidney failure in this patient underscores the potential severity of renal-predominant disease [17]. Although renal biopsy remains the diagnostic gold standard for vasculitic glomerulonephritis, it was not pursued in this case because ultrasonographic evidence of chronic parenchymal damage made histopathological confirmation unlikely to alter management [18]. This decision underscores the importance of integrating imaging findings and clinical judgment when invasive diagnostic tests confer limited benefit.

Immunosuppressive therapy with high-dose corticosteroids and cyclophosphamide was clinically indicated and resulted in rapid suppression of peripheral eosinophilia and partial stabilization of renal function [19]. At presentation, the patient’s serum creatinine was 723 µmol/L, corresponding to an eGFR of approximately 6.5 mL/min/1.73 m^2^ (CKD-EPI 2021). Following initiation of immunosuppressive therapy, renal function remained stable, with no further increase in serum creatinine levels, preserved urine output, and no immediate need for renal replacement therapy. However, despite temporary stabilization, kidney function remained within the end-stage range (eGFR <10 mL/min/1.73 m^2^), and the patient progressed to end-stage kidney disease, necessitating preparation for renal replacement therapy. The decision to create a left radiocephalic AVF was made in the context of persistently advanced renal impairment with an eGFR of approximately 6-7 mL/min/1.73 m^2^, in accordance with current nephrology practice guidelines, which do not consider active ANCA-associated vasculitis a contraindication to vascular access creation in patients anticipated to require renal replacement therapy [20]. Timely pre-dialysis planning of vascular access is routinely recommended to minimize catheter dependence and associated complications, and there is no evidence-based rationale for delaying fistula construction until complete remission of systemic inflammatory disease is achieved. Nevertheless, the potential impact of active vascular inflammation on early access integrity and maturation remains insufficiently addressed in current clinical guidance [21].

Fatal hemorrhage from AVFs is a rare but recognized complication in patients with chronic kidney disease [22]. Most reported cases involve aneurysmal degeneration, infection, skin erosion, or mechanical trauma of mature vascular access [23]. In contrast, the present case is notable for the exceptionally short interval between fistula construction and catastrophic rupture. Although a direct causal relationship cannot be definitively established, the concurrence of active systemic vasculitis, recent exposure to high-dose corticosteroids, cytotoxic immunosuppression, and underlying vascular fragility may have contributed to vascular vulnerability and impaired tissue integrity [24]. Active small- and medium-vessel vasculitis in EGPA is characterized by eosinophil-mediated endothelial injury, inflammatory cell infiltration, and fibrinoid necrosis of the vascular wall, which may impair structural integrity under conditions of increased hemodynamic stress [25]. In the early postoperative phase following AVF creation, such inflammatory alterations, compounded by corticosteroid-induced impairment of connective tissue repair and cyclophosphamide-induced inhibition of cellular proliferation, may theoretically predispose to localized weakening of the vascular wall prior to complete access maturation [26]. Additionally, although low-dose acetylsalicylic acid may have influenced bleeding severity once rupture occurred, its contribution to the structural failure of the vascular access cannot be determined from the available clinical data [27].

Importantly, the fistula was clinically unremarkable during the early postoperative period, with preserved thrill and no signs of infection or ischemia, underscoring that the absence of local warning signs does not preclude catastrophic bleeding [28]. This observation reinforces that vascular access-related mortality, although uncommon, may occur abruptly and outside the hospital setting, leaving minimal opportunity for effective intervention.

From a clinical perspective, this case emphasizes the need for individualized risk assessment when planning invasive vascular procedures in patients with active systemic vasculitis. Although fistula creation was clinically indicated in the present case, this observation raises the possibility that individualized timing of vascular access procedures in selected patients with active systemic vasculitis may warrant further clinical consideration. Equally important is structured patient and caregiver education on recognizing and immediately managing access-site bleeding, as early compression and rapid emergency response remain the only potentially life-saving measures. This may be particularly relevant during the early postoperative period and while patients are receiving high-dose immunosuppressive therapy.

This report has inherent limitations related to its single-patient design. The absence of renal histopathology precludes definitive characterization of the underlying glomerular lesion, and no postmortem examination was performed to determine the precise mechanism of fistula rupture. Consequently, a direct causal relationship between active vasculitis, immunosuppressive therapy, and vascular access failure cannot be conclusively established. Furthermore, the contribution of local surgical factors and concomitant antiplatelet therapy to the bleeding event cannot be quantified from the available clinical data.

To our knowledge, spontaneous rupture of a newly constructed AVF in the early postoperative period in a patient with renal-predominant EGPA has not been reported. Although immunosuppressive therapy resulted in hematological response and temporary stabilization of renal function, reflected by the absence of further creatinine increase and preserved urine output, treatment was initiated primarily due to organ-threatening systemic disease activity, including peripheral neuropathy, serosal involvement, and marked eosinophilia, rather than with the expectation of meaningful renal recovery in the setting of advanced chronic kidney disease (eGFR ≈6-7 mL/min/1.73 m^2^). In this context, ongoing systemic inflammatory activity may have persisted despite partial laboratory response, raising the possibility that vascular access created during periods of active systemic vasculitis may be particularly vulnerable to structural compromise, even in the absence of overt local warning signs.

## Conclusions

EGPA may present with prolonged, fragmented manifestations that delay diagnosis until irreversible organ damage has occurred. Renal-predominant disease can progress rapidly and necessitate timely preparation for renal replacement therapy in accordance with standard nephrology practice. This case highlights that, although AVF creation is not contraindicated in patients with active ANCA-associated vasculitis, vascular access procedures performed during periods of active systemic inflammation may carry a rare but potentially fatal risk of early access failure and catastrophic hemorrhage. Recognition of this potential vulnerability may be clinically relevant when planning invasive procedures in patients with active multisystem inflammatory disease; however, a causal relationship between active systemic inflammation and early vascular access failure cannot be established based on the findings of this single case and remains speculative.
